# Narrowing Ratio of Retinal Veins at Arteriovenous Crossing in Patients With Branch Retinal Vein Occlusion Versus That in Healthy Individuals

**DOI:** 10.1167/iovs.64.14.22

**Published:** 2023-11-16

**Authors:** Ryo Tomita, Kensuke Goto, Yoshitaka Ueno, Katsuya Yamaguchi, Jun Takeuchi, Tomohiko Akahori, Hiroki Kaneko, Takeshi Iwase

**Affiliations:** 1 Department of Ophthalmology, Nagoya University Graduate School of Medicine, Showa-ku, Nagoya, Aichi, Japan; 2 Department of Ophthalmology, Akita University Graduate School of Medicine, Akita, Japan

**Keywords:** branch retinal vein occlusion (BRVO), optical coherence tomography (OCT), retinal vein, arteriovenous crossing

## Abstract

**Purpose:**

This cross-sectional study aimed to clarify the differences in the retinal venous narrowing ratio (VNR) at retinal arteriovenous crossing by optical coherence tomography (OCT) among the eyes with branch retinal vein occlusion (BRVO), fellow eyes of patients with BRVO, and eyes of individuals without BRVO and to determine factors that influence the VNR.

**Methods:**

We studied 31 eyes of young participants, 54 eyes of an older control group, 56 fellow eyes of patients with BRVO, and 48 eyes with BRVO. Cross-sectional OCT images were used to determine the VNR at two arteriovenous crossings per eye.

**Results:**

Overall, 378 arteriovenous crossings were analyzed. The VNR of arterial overcrossings of fellow eyes (27.7% ± 11.1%) and BRVO eyes (27.3% ± 9.76%) were significantly higher than those in the young (16.0% ± 7.9%, all *P* < 0.001) and control (22.0% ± 8.81%, *P* < 0.001, *P* = 0.003, respectively) groups. The VNR of arterial overcrossings was significantly larger than that of venous overcrossings (24.0% ± 10.5% vs. 20.6% ± 13.0%, *P* = 0.021). A linear mixed-effects model showed that the VNR was significantly higher in arterial overcrossings, crossings with larger arterial internal diameters, smaller venous internal diameters, and participants with older age and a BRVO history.

**Conclusions:**

The VNR in arterial overcrossings was higher in BRVO eyes and even in the fellow eyes. Thus, a higher VNR in arterial overcrossings may contribute to BRVO development, and crossings with factors contributing to higher VNR might be associated with a risk of BRVO.

A branch retinal vein occlusion (BRVO) is a relatively common retinal vascular disorder with an incidence of 0.3% to 2.0%.^1^^–^^4^ Color fundus photograph analysis revealed that most BRVO develops at arteriovenous crossings with a retinal artery crossing over a retinal vein (arterial overcrossing)^5^^,^^6^ because the vein deviates into the vitreous cavity and decompresses at arteriovenous crossings with a retinal vein crossing over a retinal artery (venous overcrossing).^5^^,^^7^^–^^9^

However, recent studies of optical coherence tomography (OCT) images have shown that BRVO more frequently occurs at venous overcrossings, compared with earlier studies using color fundus photographs.^10^^–^^12^ According to Muraoka et al., mechanical compression caused by the arterial wall and retina at the arterial overcrossing might be less important in the pathogenesis of BRVO, and the mechanical compression caused by the arterial wall with the internal limiting membrane (ILM) in the venous overcrossing might be important.^12^ However, this study was conducted in crossings after the development of BRVO and might be influenced by secondary effects, such as blood flow disturbances caused by BRVO. Moreover, no recent reports have compared the differences in the degree of venous narrowing at arteriovenous crossings between arterial and venous overcrossings without BRVO or between the eyes of patients with BRVO and those of non-BRVO controls. Consequently, whether the degree of venous narrowing varies by the crossing pattern or whether the degree of venous narrowing is higher in eyes at high risk for BRVO remains unclear in arteriovenous crossings without BRVO. Thus, this study discerned the characteristics of the degree of venous narrowing at the arteriovenous crossing in several groups. The degrees of venous narrowing were compared among the fellow eyes of patients with BRVO, BRVO eyes, and control eyes, and the factors associated were addressed.

## Methods

### Ethics Statement

This observational cross-sectional study was approved by the Ethics Committee of Nagoya University Hospital, and the procedures conformed to the tenets of the Declaration of Helsinki. Informed consent was obtained from all participants.

### Subjects and Testing Protocol

This study was conducted at the Nagoya University Hospital between January 2017 and April 2020. Individuals with two arterial or venous overcrossings per eye, that is, one in the superior retina and the other in the inferior retina, were included. These findings were made from the analyses of spectral-domain OCT images.

The eyes were divided into four groups. The first group (young group) consisted of normal eyes of healthy young adult volunteers aged 20 to 40 years without any ocular or systemic diseases. The second group (control group) included healthy eyes of individuals aged >45 years without any retinal diseases. The third group consisted of the fellow eyes of the BRVO group (fellow eye group), in which none had any retinal diseases. The fourth group consisted of eyes with BRVO without other retinal diseases (BRVO eye group). The presence of an ischemia area was determined based on the results of OCT angiography or fundus fluorescein angiography. Ischemia was defined as an area without perfusion of more than five optic discs. Details of the BRVO eyes are given in Supplementary Table S1. Most BRVO eyes had received treatment with intravitreal injections of anti-vascular endothelial growth factor (anti-VEGF) agents, sub-Tenon injection of triamcinolone acetonide, or retinal laser photocoagulation. Treatments were not standardized and were given according to the physician. As described later, arteriovenous crossings in the BRVO eye group were selected from areas that were not directly affected by BRVO; however, they might be affected by certain blood flow abnormalities due to BRVO or its treatment. Therefore, crossings in the fellow eye group were regarded as the BRVO high-risk eye because studies have reported that BRVO developed in the fellow eye of the BRVO patients at approximately 5% to 10%,^13^^–^^19^ which is relatively higher than the general incidence.^1^^–^^4^

History of hypertension, hyperlipidemia, diabetes, and smoking were obtained from the interview records. All patients underwent comprehensive ophthalmologic examination, including measurements of intraocular pressure (IOP), slit-lamp biomicroscopy, indirect ophthalmoscopy, OCT (Spectralis; Heidelberg Engineering, Heidelberg, Germany), and blood pressure (BP), which was measured with an automatic sphygmomanometer (CH-483C; Citizen, Tokyo, Japan) for screening other ocular diseases. Mean BP and mean ocular perfusion pressure (OPP) were calculated by the following formulas:

$$\begin{array}{ccc} & & Mean\;BP=diastolic\;BP+1/3\;\left(systolic\;BP-diastolic\;BP\right)\\ & & Mean\;OPP=2/3*\;Mean\;BP-IOP \end{array}$$

### Exclusion Criteria

Eyes were excluded if they had prior vitreous surgeries and severe ocular media opacities, including severe cataracts, vitreous opacities, or retinal disease (e.g. macular degeneration, epiretinal membrane, and diabetic retinopathy). Eyes were also excluded if they had arteriovenous crossings that met the following conditions: not present in either the superior or inferior retina of the same eye and the venous narrowing ratio (VNR) could not be calculated because the center of the arteriovenous crossing was not recorded. BRVO eyes that had received any treatment (e.g. intravitreal injections of anti-VEGF agents, sub-Tenon's capsule injection of triamcinolone acetonide, or retinal laser photocoagulation) within 1 month of study initiation were also excluded.

### Examinations of Arteriovenous Crossings

According to the rules described below, two retinal arteriovenous crossings were selected based on the fundus photograph or infrared OCT images, with one from the superior and one from the inferior hemisphere. The crossings closest to the optic disc were selected; however, they must be within an area three times the optic disc's diameter. The crossings were also unselected if a vein branch was close to the crossing. An artery and a vein were differentiated by cross-referencing the fundus photograph, the infrared OCT image, and the B scan of OCT. This selection does not consider whether the crossing is an arterial or venous overcrossing. In the BRVO eye group, arteriovenous crossings were selected outside of the BRVO area, that is, outside of areas with past or present hemorrhage or edema. Cross-sectional OCT images of the arteriovenous crossings, retinal arteries, and veins were recorded in 25 or 49 sections of 15 degrees × 5 degrees, and the image from each section was the average of 50 scans.

**Figure 1.**
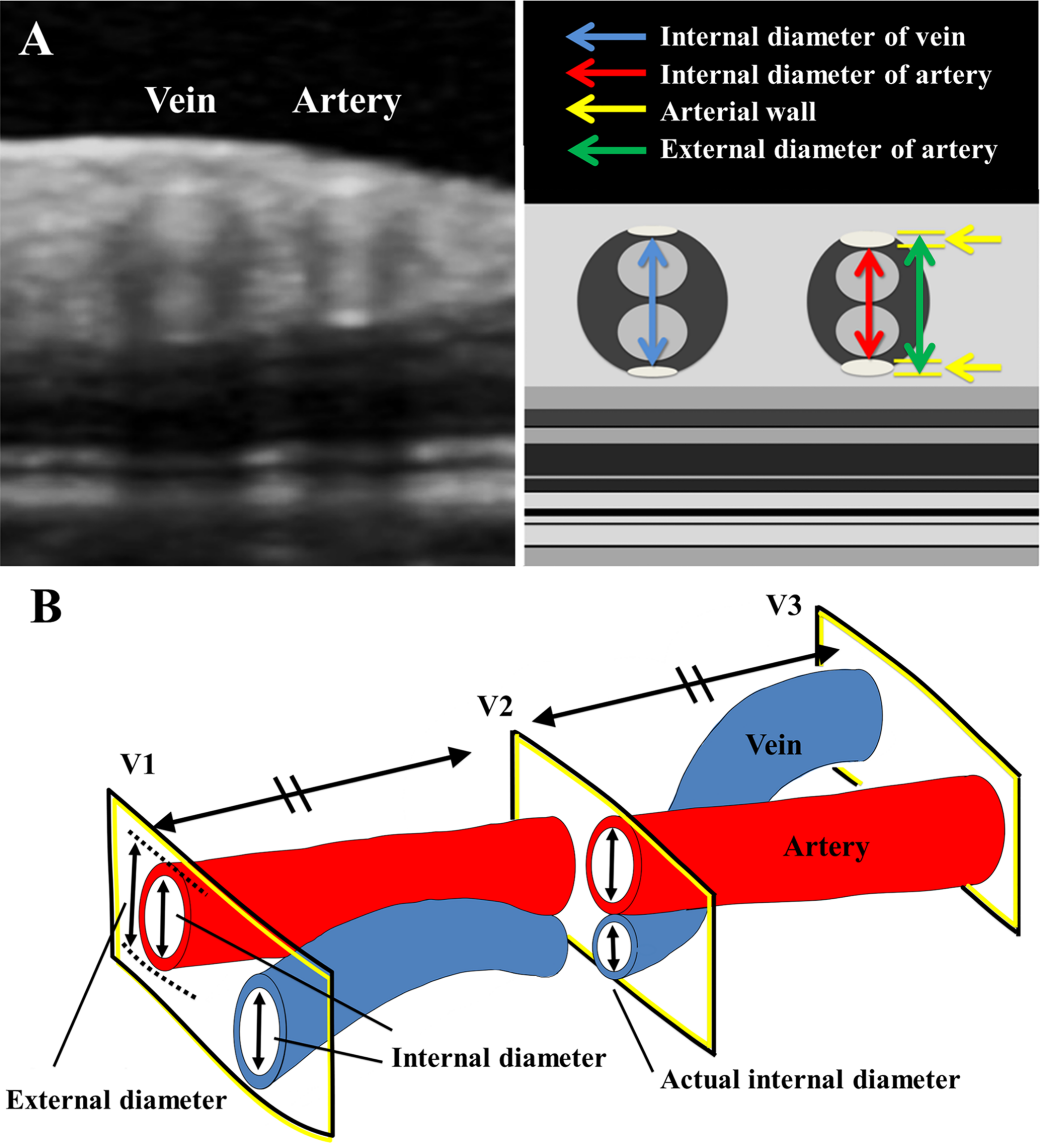
Measurement of retinal vessels by optical coherence tomography (OCT). (**A**) Cross-sectional OCT image and schematic diagram of the retinal artery and vein. In both images, the vein is on the *left*, and the artery is on the *right*. The two high intensities at the *top* and *bottom* indicated the vessel walls, and those in the *middle* indicate the presence of blood flow. The *blue arrow* indicates the internal diameter of the vein; the *red arrow* indicates the internal diameter of the artery; the *green arrow* indicates the external diameter of the artery; and the *yellow arrow* indicates the arterial wall thickness. (**B**) Schematic diagram of the method for measuring the venous narrowing ratio (VNR). The average of the vessel diameters measured before and after the crossing (V1 and V3) was defined as the calculated diameter. The diameter measured at the crossing (V2) was defined as the actual diameter. The VNR was obtained using the following formula: VNR = (calculated internal diameter of vein – actual internal diameter of vein)/calculated internal diameter of vein × 100.

Several studies have evaluated retinal blood vessels and arteriovenous crossings using OCT images.^12^^,^^20^^–^^23^ Reproducibility of retinal vessel diameter measurements from OCT images^24^^–^^26^ and significant correlations with measurements from adaptive optics scanning laser ophthalmoscopy (AOSLO) have been reported.^21^ In OCT images, retinal blood vessels have four hyperreflective patterns in a circular hyporeflective configuration (Fig. 1A). The two outermost hyper-reflective patterns indicate the vessel walls.^21^ In most cases, the vessel wall appears only as a high reflection where the OCT projection light is projected perpendicular to the vessel wall.^24^ The perpendicular distance between the inner edges of the two hyper-reflective lines is considered the internal diameter of the retinal blood vessels. The perpendicular distance between the outer edges of the two hyper-reflective lines is considered the external diameter.^21^^,^^22^ The diameters were measured at three points, before and after the crossing, and at the crossing using the caliper tool on the software embedded in the OCT. The diameters before and after the crossing were measured at approximately equal distances on the retinal plane from the crossing to each. The diameter at the crossing was measured only when the arterial and venous vessel walls were aligned perpendicularly. By averaging the internal diameters of the retinal vein before and after the arteriovenous crossing, the calculated internal diameter of the vein at the arteriovenous crossing was obtained. The VNR can be obtained by comparing the calculated internal diameter of the vein with the actual venous internal diameter at the arteriovenous crossing (Fig. 1B).^22^ The arterial wall thickness to the lumen diameter ratio (AWLR) of the retina was defined as a structural parameter of the microvascular damage, as reported.^27^^–^^29^

The VNR and AWLR were computed according to the following formulas:

$$\begin{array}{c} \begin{array}{c} Venous\;narrowing\;ratio\;(VNR)=(calculated\;internal\\ \;diameter\;of\;the\;vein-actual\;internal\;diameter\;of\;the\\ \;vein\;at\;the\;crossing\;site)/calculated\;internal\;diameter\\ \;of\;the\;vein\times 100\;(\%)\\ Arterial\;wall\;to\;lumen\;ratio=(calculated\;external\\ \;diameter\;of\;the\;artery-calculated\;internal\;diameter\\ \;of\;the\;artery)/calculated\;internal\;diameter\\ \;of\;the\;artery \end{array} \end{array}$$

Two graders independently measured vessel diameters on OCT scans at 100 randomly selected crossings, and the intraclass correlation coefficient (ICC) was used to determine the reproducibility of the VNR measurement.

### Statistical Analyses

The Shapiro-Wilk test was utilized to evaluate the normality of variable distributions, and Levene's test was used to test the equality of variances. In analyzing participant characteristics, analysis of variance was used for variables that were normally distributed and showed equality of variance, and Tukey's test for between-group comparisons was used. In the case of the other variables, Kruskal-Wallis test and Dunn's test with Bonferroni correction were done. History of systemic diseases such as hypertension was evaluated with a chi-square test, followed by pairwise comparisons with Bonferroni correction. Linear mixed-effect (LME) models, with patients as the random effect, were used to compare the measured OCT parameters among the four groups, the VNRs of arterial overcrossing and venous overcrossings, and to analyze for factors affecting the VNR. Tukey's correction was used to adjust for multiple comparisons. The LME model is similar to ordinary linear regression. However, all observations are assumed to be independent of each other in an ordinary linear regression model. However, the measurements used in this study were nested within participants and arteriovenous crossings, which render them interdependent. The neglect of this grouping of measurements would have resulted in an underestimation of the standard error of the regression coefficients. To address this concern, we utilized an LME model that accounts for the hierarchical structure of the data and incorporates within-subject measurements to minimize the potential bias that may arise from the nested data structure.

In the LME model for factors affecting the VNR, the young group was excluded to reduce selection bias. The BRVO eye group was also excluded because they may have been affected by BRVO or BRVO treatment, and about half of the fellow eye and BRVO eye groups were the eyes of the same patients with BRVO. Data are presented as the mean ± standard deviation of the means. A *P* value < 0.05 was taken to be significant. The sample size was estimated, aiming to compare 4 groups with an assumed standard deviation of 10 in the VNR with reference to the previous study.^22^ Targeting a 5% mean difference, with a significance level of 0.05 and 80% power, we estimated a required sample size of approximately 97 crossings per group. All statistical analyses were performed using the statistical programming language R. version 4.2.2 (The R Foundation for Statistical Computing, Vienna, Austria).

## Results

This study analyzed 378 arteriovenous crossings in 189 eyes of 156 individuals. The mean age of the participants was 62.1 ± 17.5 years, with a range of 22–84 years. Table 1 shows the clinical and demographic data of the participants in each group. The fellow eye group had significantly shorter axial lengths than the young group (*P* < 0.001) and the control group (*P* = 0.018), and the BRVO eye group also had shorter axial lengths than the young group (*P* = 0.003). The young group had significantly younger age (all, *P* < 0.001) and lower mean BP (*P* = 0.002, *P* < 0.001, and *P* < 0.001, respectively), mean ocular perfusion pressure (*P* = 0.012, *P* < 0.001, and *P* < 0.001, respectively), and fewer patients with history of hypertension (*P* = 0.008, *P* < 0.001, and *P* < 0.001, respectively) than in the control, fellow eye, and BRVO eye groups. Fewer patients in the young and control groups had a history of dyslipidemia than in the BRVO eye group (*P* = 0.002 and *P* = 0.049, respectively). Mean OPP in the BRVO eye group was higher than that in the control group (*P* = 0.034). No other significant differences were found among the three older groups.

**Table 1.** Summary of the Clinical and Demographic Data of the Participants

	All Groups	Young Group	Control Group	Fellow Eye Group	BRVO Eye Group	*P* Value
No of eyes/crossings	189/378	31/62	54/108	56/112	48/96	—
Sex, M/F	108/81	14/17	29/25	35/21	30/18	0.952
Age, y	62.1 ± 17.5	28.3 ± 3.60	68.0 ± 8.50^*^	69.1 ± 10.0^*^	69.0 ± 10.6^*^	<0.001
Axial length, mm	24.3 ± 1.50	25.1 ± 1.36	24.7 ± 1.76	23.7 ± 1.21^*^^,^^†^	23.9 ± 1.16^*^	<0.001
IOP, mm Hg	13.4 ± 3.13	12.7 ± 2.90	14.2 ± 2.83	13.4 ± 3.09	12.9 ± 3.47	0.117
Mean BP, mm Hg	98.6 ± 16.3	85.9 ± 11.7	98.2 ± 15.9^*^	101 ± 14.5^*^	104 ± 17.3^*^	<0.001
Mean OPP, mm Hg	52.3 ± 10.4	44.5 ± 7.59	51.3 ± 9.85^*^	53.9 ± 9.13^*^	56.6 ± 11.1^*^^,^^†^	<0.001
Hypertension	70	0	17^*^	27^*^	26^*^	<0.001
Dyslipidemia	38	0	7	13	18^*^^,^^†^	<0.001
Diabetes mellitus	18	0	5	6	7	0.188
History of smoking	44	2	15	17	10	0.06

BRVO, branch retinal vein occlusion; BP, blood pressure; IOP, intraocular pressure; OPP, ocular perfusion pressure.

* Significant difference compared to the young group;

† Significant difference compared to the control group.

**Figure 2.**
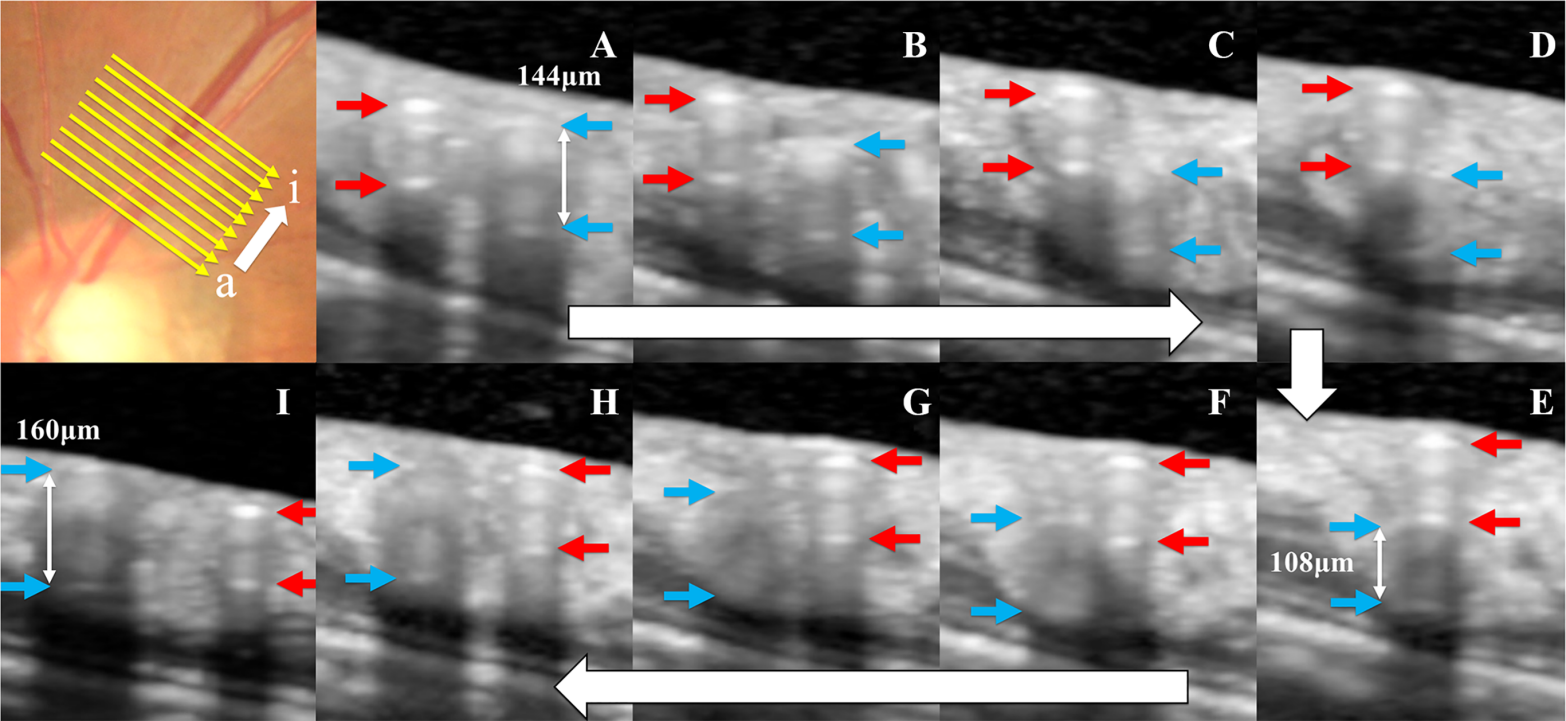
Representative case of an arterial overcrossing. Optical coherence tomography (OCT) images showing the arterial overcrossing pattern in the superior retina of the fellow eye of a female patient with branch retinal vein occlusion (BRVO) and history of hypertension. The lumen of the vein appeared not compressed but narrowed beneath the artery at the crossing. The internal diameters of the vein on the optic disc side, of the vein at the crossing, and of the vein distal to the crossing were 144, 108, and 160 µm, respectively, and the venous narrowing ratio was 28.9%.

**Figure 3.**
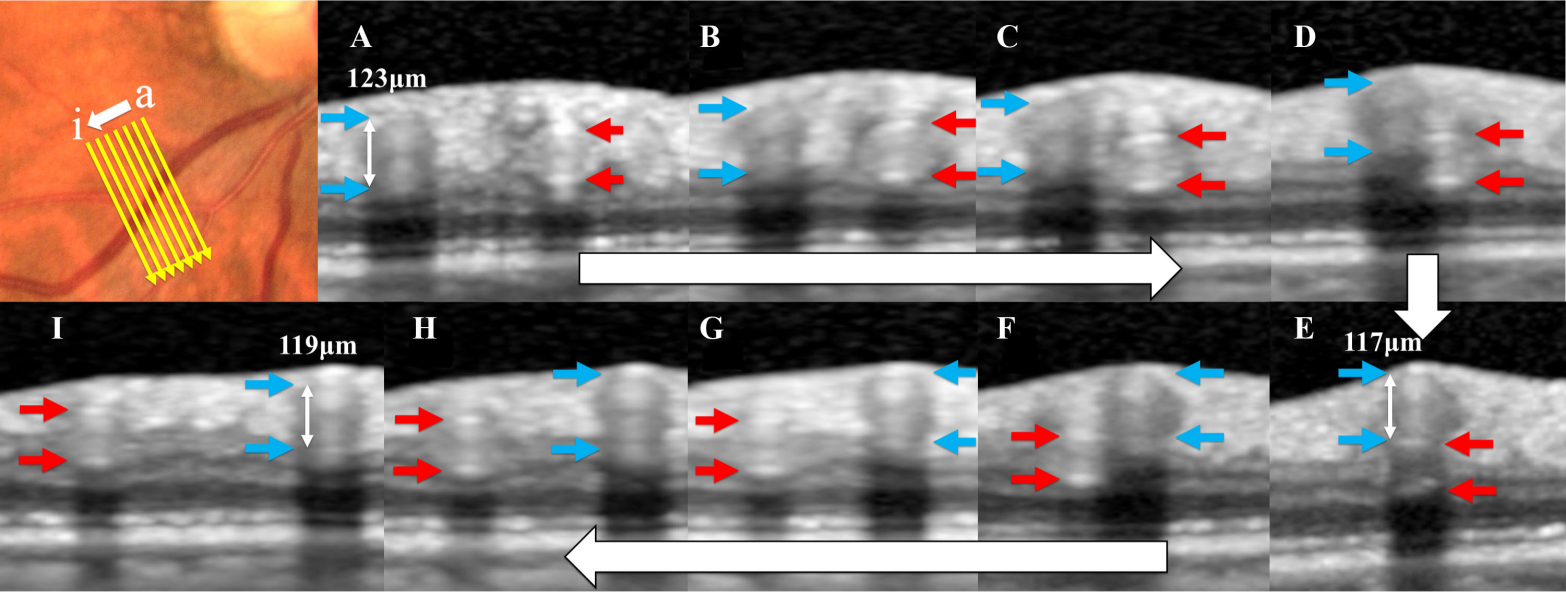
Representative case of a venous overcrossing. Optical coherence tomography (OCT) images showing the venous overcrossing pattern in the inferior retina of the fellow eye of a female patient with branch retinal vein occlusion (BRVO) and history of hypertension. The vein protruded toward the vitreous side, and the lumen of the vein appeared to be maintained at the crossing. The internal diameters of the vein on the optic disc side, of the vein at the crossing, and of the vein distal to the crossing were 123, 117, and 119 µm, respectively, and the venous narrowing ratio was 3.3%.

Figures 2 and 3 show representative OCT images of arterial and venous overcrossings. The internal diameters of the vein in both crossing patterns were not compressed and remained circular in all crossings. The parameters of the blood vessels measured by OCT are shown in Supplementary Table S2. The control, fellow, and BRVO eye groups had significantly higher AWLR than the young group (*P* = 0.043, *P* = 0.002, and *P* = 0.016, respectively).

**Figure 4.**
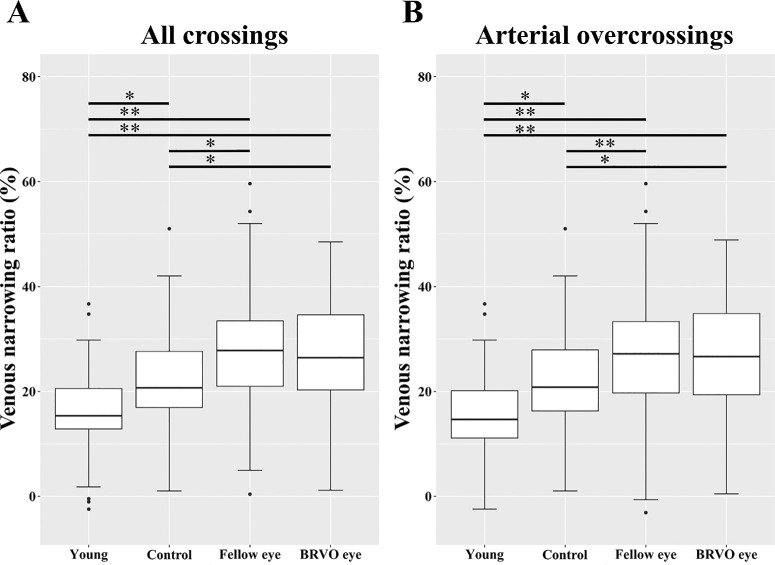
Differences in the venous narrowing ratio (VNR) of the four groups. (**A**) In all crossings, the VNR was significantly lower in the young group than in the control, fellow, and branch retinal vein occlusion (BRVO) eye groups (*P* = 0.001, *P* < 0.001, and *P* < 0.001, respectively), and significantly higher in the fellow and BRVO eye groups than in the control group (*P* = 0.005 and *P* = 0.005, respectively). (**B**) In the arterial overcrossing, the VNR was significantly lower in the young group than in the control, fellow, and BRVO eye groups (*P* = 0.003, *P* < 0.001, and *P* < 0.001, respectively) and significantly higher in the fellow and BRVO eye groups than in the control group (*P* < 0.001 and *P* = 0.003, respectively). **P < 0.001 and *P < 0.01.

Table 2 shows the mean VNR in each crossing pattern and group. In all crossings, the VNR was significantly lower in the young group (15.5 ± 7.61%) than in the control (21.8 ± 9.02%, *P* = 0.001), fellow (26.5 ± 12.1%, *P* < 0.001), and BRVO eye groups (26.7 ± 11.0%, *P* < 0 .001) and significantly higher in the fellow (*P* = 0.005) and BRVO eye groups (*P* = 0.005) than in the control group (Fig. 4A). In arterial overcrossings, the VNR was significantly lower in the young group (16.0 ± 7.94%) than in the control (22.0 ± 8.81%, *P* = 0.003), fellow (27.7 ± 11.1%, *P* < 0.001), and BRVO eye groups (27.3 ± 9.76%, *P* < 0.001) and significantly higher in the fellow (*P* < 0.001) and BRVO eye groups (*P* = 0.003) than in the control group (Fig. 4B). No significant difference in the VNR of venous overcrossings was found among the four groups. In 378 arteriovenous crossings, 309 (81.7%) were arterial overcrossings and 69 (18.3%) were venous overcrossings. The VNR in arterial overcrossings (24.0 ± 10.5%) was higher than that in venous overcrossings (20.6 ± 13.0%, *P* = 0.021). The ICC of the VNR measurements by two graders was 0.91 (95% confidence interval = 0.87 − 0.94).

**Table 2.** Venous Narrowing Ratio of Each Group

Pattern of Crossing	Group	No. of Crossings	Venous Narrowing Ratio, %
All crossings	Young eyes	62	15.5 ± 7.61
	Control eyes	108	21.8 ± 9.02
	Fellow eye of the BRVO eye	112	26.5 ± 12.1
	BRVO eye	96	26.7 ± 11.0
	All groups	378	23.4 ± 11.1
Arterial overcrossing	Young eyes	51	16.0 ± 7.94
	Control eyes	89	22.0 ± 8.81
	Fellow eye of the BRVO eye	95	27.7 ± 11.1
	BRVO eye	74	27.3 ± 9.76
	All groups	309	24.0 ± 10.5
Venous overcrossing	Young eyes	11	13.1 ± 5.52
	Control eyes	19	21.1 ± 10.2
	Fellow eye of the BRVO eye	17	20.0 ± 15.8
	BRVO eye	22	24.5 ± 14.4
	All groups	69	20.6 ± 13.0

BRVO, branch retinal vein occlusion.

The results of the LME model for the effects on the VNR were shown in Table 3. Arterial overcrossing (β = 4.00, *P* = 0.034), BRVO history in the opposite eye (β = 5.71, *P* < 0.001), calculated internal diameter of the artery (β = 0.175, *P* = 0.007), and age (β = 0.209, *P* = 0.011) had significant positive effects on the VNR, and the calculated internal diameter of the vein had a significant negative effect (β = −0.091, *P* = 0.006). The location of the crossings in the inferior retina also had a negative but not significant effect (β = −2.54, *P* = 0.064).

**Table 3.** Linear Mixed-Effect Model for Effects on the Venous Narrowing Ratio

Coefficient	β	95% CI	*P* Value
Intercept	−23.1	−55.2 to 8.93	0.237
Crossing type (1 = arterial overcrossing)	4.00	0.916 to 7.09	0.034
BRVO history (1 = presence of BRVO in the opposite eye)	5.71	3.33 to 8.09	<0.001
Calculated internal diameter of the vein	−0.091	−0.145 to −0.036	0.006
Calculated internal diameter of the artery	0.175	0.068 to 0.281	0.007
Arterial wall to lumen ratio	−0.805	−23.2 to 21.6	0.953
Age	0.209	0.075 to 0.343	0.011
Location of the crossings (1 = inferior retina)	−2.54	−4.79 to −0.296	0.064
Mean ocular perfusion pressure	−0.014	−0.136 to 0.107	0.847
Axial length	1.04	0.147 to 1.93	0.057

95% CI, 95% confidence interval; BRVO, branch retinal vein occlusion.

## Discussion

This study showed that VNRs in the fellow and BRVO eye groups were significantly higher than that in the control group. The LME model showed that the VNR was higher in arterial overcrossings, crossings with larger arterial internal diameters, smaller venous internal diameters, and in participants with older age and a BRVO history.

In this study, the mean VNR at all crossings was 23.4%. This ratio is comparable with those in previous reports.^22^ Although this analysis of factors that affect the VNR would be ideally performed on BRVO eyes, the interpretation of the results is difficult because BRVO eyes are likely to be affected by blood flow disturbances caused by BRVO or treatment. Therefore, we excluded BRVO eyes from the LME model to analyze their effect on the VNR. Because the high incidence of BRVO in the fellow eyes has already been reported, we regard the fellow eyes as high-risk eyes for BRVO.^13^^–^^19^ The young group had lower VNR than the other groups, and age was also significantly positively correlated with the VNR in the LME model performed in the control and fellow eye groups. This result agrees with the higher incidence of BRVO in the aged populations.^30^ It is also reasonable that the VNR is higher at crossings with larger arteries and smaller veins. In addition, the crossing in the superior retina had a 2.4% higher VNR than the crossing in the inferior retina, although it was not significant. With regard to the difference between the superior and inferior retina, it has been shown that BRVO is found more frequently in the superior retina than in the inferior retina^31^ and that blood flow and vessel diameter in the superior retina is greater than in the inferior retina.^32^ The difference in blood flow between the superior and inferior retina could cause differences in venous narrowing, leading to the difference in the frequency of BRVO.

Recent studies using OCT for crossings with BRVO suggested that mechanical compression at arterial overcrossings may be less important in the pathogenesis of BRVO.^12^ In this study, although none of the crossing patterns resulted in the compression of the lumen, the VNR in arterial overcrossings was higher in the fellow eye group than in the control eye group. The results of previous and current studies agree with each other. In other words, although compression is not the cause of BRVO development in arterial overcrossings, narrowing may lead to BRVO development. The narrowing of the venous lumen at crossings increases venous flow velocity and pressure, further damaging the venous endothelium. Histological studies have shown focal stratification of the venous basement membrane at arteriovenous crossings and elongation of venous endothelial cells.^33^^,^^34^ This endothelial dysfunction possibly contributes to venous thrombosis. Thus, even if the vein is not compressed, the narrower vein at the crossing may increase the incidence of BRVO. This result suggests that a high narrowing ratio is a risk for BRVO development, even in arterial overcrossings.

The higher VNR, even in the fellow eye group, may be due to the characteristics of the vessels in patients with BRVO. Increased arterial stiffness and a higher incidence of cardiovascular and cerebrovascular events have been reported in patients with BRVO.^35^^–^^37^ These vascular characteristics in patients with BRVO may affect the VNR. Although the effect of the AWLR on the VNR was not significant in this study, it may have been affected by the difference in the accuracy between the lumen and arterial wall measurements in OCT. Retinal vessel diameter measurements by OCT images were highly reproducible and correlated with measurements obtained by AOSLO.^21^^,^^24^ However, retinal arterial wall thicknesses measured by OCT and AOSLO were reported to have no significant correlation.^21^ Investigating factors that affect the VNR using other testing instruments may lead to new findings, especially concerning arterial wall thickness.

As in the representative cases shown in Figure 3, the internal diameter of the vein at a venous overcrossing was maintained and not compressed. Moreover, the VNR was smaller in a venous overcrossing than in an arterial overcrossing. This result agreed with previous studies reporting that the vein in venous overcrossings was unlikely to narrow.^7^^–^^9^^,^^34^ However, it is inconsistent with the finding of a previous study using OCT, which revealed that the venous lumen in a venous overcrossing with BRVO was compressed.^12^^,^^22^ This inconsistency in venous overcrossings appears to be due to the difference in the status of the crossings studied, that is, the analyzed crossings developed BRVO in the previous OCT study, whereas current crossings had not developed BRVO. A possible explanation is that the venous lumen at the venous overcrossing in the normal state may be less susceptible to narrowing than the arterial overcrossing. In cases where the venous lumen at venous overcrossing is severely narrowed because of increased pressures from the vitreous cavity, ILM, or retinal artery, BRVO may develop. In addition, the results of the previous study by OCT may have been affected by damage to the retinal vessels or blood flow disturbance caused by BRVO. Another possibility is that arterial movement with the pulse may cause mechanical damage independent of venous compression because the artery and vein are tethered together at a crossing. However, few studies have focused on the cause of venous narrowing during venous overcrossing. Further research is needed to identify the causes, and the mechanisms for developing BRVO during venous overcrossing may be determined.

This study had several limitations. First, the diameter of the venous lumen was measured only in the vertical direction of the retina. The vessel walls of the vertical sides can be well viewed in OCT images; however, those of the horizontal sides cannot be viewed well. On this point, AOSLO can measure the vessel walls on the horizontal side; however, measuring lumens in crossing by AOSLO is not feasible due to the overlying vessels. Second, we excluded crossings in which the veins divided immediately before or after the crossings. These crossings might be associated with BRVO development; however, VNR cannot be calculated in these crossings. Third, the BRVO eye group included eyes that had been previously treated; hence, it was excluded while analyzing factors that affect the VNR because they may have been affected by treatment or BRVO. As a result, the VNR in the BRVO eye group was almost the same as that in the fellow eye group. Fourth, we focused on the arteriovenous crossings where no BRVO had occurred and not on the crossings where BRVO had occurred. Therefore, our study might be different when examining the actual conditions of BRVO. However, the crossing after BRVO onset is affected by subsequent changes in the retinal vessel and blood flow because of thrombosis, a change in the perfusion status of the retina, VEGF, or various inflammatory cytokines. Fifth, the axial length of the eye was different between the eyes of patients with BRVO and the young and control eyes. This may be because the axial lengths of BRVO eyes are relatively short.^38^^–^^40^ Future studies with controls adjusted for ocular axis may be preferable, but the eye with a short ocular axis may itself be a risk factor. Because it is reported that axial scaling is independent of ocular magnification in OCT images,^41^ we did not correct measurements by ocular axis length in this study.

In conclusion, the VNR was higher in the BRVO eyes and even fellow eyes. The VNR was higher in arterial overcrossings, crossings with larger arterial internal diameters, smaller venous internal diameters, and participants with older age and a BRVO history. These findings suggest that higher VNR in arterial overcrossings may contribute to BRVO development and that crossings with factors contributing to higher VNR might be associated with a risk of BRVO.

## Supplementary Material

Supplement 1

## References

[1] Wong TY, Larsen EK, Klein R, et al. Cardiovascular risk factors for retinal vein occlusion and arteriolar emboli: the atherosclerosis risk in communities & cardiovascular health studies. *Ophthalmology*. 2005; 112: 540–547. 15808241 10.1016/j.ophtha.2004.10.039

[2] Li JQ, Terheyden JH, Welchowski T, et al. Prevalence of retinal vein occlusion in Europe: a systematic review and meta-analysis. *Ophthalmologica*. 2019; 241: 183–189. 30517942 10.1159/000494224

[3] Keel S, Xie J, Foreman J, van Wijngaarden P, Taylor HR, Dirani M. Prevalence of retinal vein occlusion in the australian national eye health survey. *Clin Exp Ophthalmol*. 2018; 46: 260–265. 28752913 10.1111/ceo.13031

[4] Arakawa S, Yasuda M, Nagata M, et al. Nine-year incidence and risk factors for retinal vein occlusion in a general Japanese population: the hisayama study. *Invest Ophthalmol Vis Sci*. 2011; 52: 5905–5909. 21693603 10.1167/iovs.11-7775

[5] Weinberg D, Dodwell DG, Fern SA. Anatomy of arteriovenous crossings in branch retinal vein occlusion. *Am J Ophthalmol*. 1990; 109: 298–302. 2309862 10.1016/s0002-9394(14)74554-4

[6] Zhao J, Sastry SM, Sperduto RD, Chew EY, Remaley NA. Arteriovenous crossing patterns in branch retinal vein occlusion. the eye disease case-control study group. *Ophthalmology*. 1993; 100: 423–428. 8460014 10.1016/s0161-6420(93)31633-7

[7] Duker JS, Brown GC. Anterior location of the crossing artery in branch retinal vein obstruction. *Arch Ophthalmol*. 1989; 107: 998–1000. 2751472 10.1001/archopht.1989.01070020060029

[8] Waisbren EC, Salz DA, Brown MM, Brown GC. Vascular crossing patterns in patients with systemic arterial hypertension. *Br J Ophthalmol*. 2013; 97: 781–784. 23603484 10.1136/bjophthalmol-2013-303100

[9] Seitz R. *The retinal vessels**.* St. Louis, MO: CV Mosby Co; 1964: 57–61.

[10] Kogo T, Muraoka Y, Iida Y, et al. Angiographic risk features of branch retinal vein occlusion onset as determined by optical coherence tomography angiography. *Invest Ophthalmol Vis Sci*. 2020; 61: 8. 10.1167/iovs.61.2.8 PMC7324438 32031580

[11] Iida Y, Muraoka Y, Ooto S, et al. Morphologic and functional retinal vessel changes in branch retinal vein occlusion: an optical coherence tomography angiography study. *Am J Ophthalmol*. 2017; 182: 168–179. 28837791 10.1016/j.ajo.2017.08.004

[12] Muraoka Y, Tsujikawa A, Murakami T, et al. Morphologic and functional changes in retinal vessels associated with branch retinal vein occlusion. *Ophthalmology*. 2013; 120: 91–99. 22980743 10.1016/j.ophtha.2012.06.054

[13] Rogers SL, McIntosh RL, Lim L, et al. Natural history of branch retinal vein occlusion: an evidence-based systematic review. *Ophthalmology*. 2010; 117: 1094–1101.e1095. 20430447 10.1016/j.ophtha.2010.01.058

[14] Glacet-Bernard A, Coscas G, Chabanel A, Zourdani A, Lelong F, Samama MM. Prognostic factors for retinal vein occlusion: a prospective study of 175 cases. *Ophthalmology*. 1996; 103: 551–560. 8618752 10.1016/s0161-6420(96)30653-2

[15] Hayreh SS, Rojas P, Podhajsky P, Montague P, Woolson RF. Ocular neovascularization with retinal vascular occlusion-III: incidence of ocular neovascularization with retinal vein occlusion. *Ophthalmology*. 1983; 90: 488–506. 6192376 10.1016/s0161-6420(83)34542-5

[16] Group BO. Argon laser photocoagulation for macular edema in branch vein occlusion: reply. *Am J Ophthalmol*. 1985; 99: 219. 10.1016/0002-9394(85)90249-1 4038586

[17] Michels R. The natural course of retinal branch vein obstruction. *Trans Am Acad Ophthalmol Otolaryngol*. 1974; 78: 166–177. 4825042

[18] Mitchell P, Smith W, Chang A. Prevalence and associations of retinal vein occlusion in Australia: the blue mountains eye study. *Arch Ophthalmol*. 1996; 114: 1243–1247. 8859084 10.1001/archopht.1996.01100140443012

[19] Branch Vein Occlusion Study Group . Argon laser scatter photocoagulation for prevention of neovascularization and vitreous hemorrhage in branch vein occlusion. A randomized clinical trial. *Arch Ophthalmol*. 1986; 104: 34–41. 10.1001/archopht.1986.01050130044017 2417579

[20] Seidel G, Aschinger G, Singer C, et al. Estimating retinal blood flow velocities by optical coherence tomography. *JAMA Ophthalmol*. 2016; 134: 1104–1110. 27490674 10.1001/jamaophthalmol.2016.2507

[21] Arichika S, Uji A, Ooto S, Muraoka Y, Yoshimura N. Comparison of retinal vessel measurements using adaptive optics scanning laser ophthalmoscopy and optical coherence tomography. *Jpn J Ophthalmol*. 2016; 60: 166–171. 26902975 10.1007/s10384-016-0435-3

[22] Kumagai K, Tsujikawa A, Muraoka Y, et al. Three-dimensional optical coherence tomography evaluation of vascular changes at arteriovenous crossings. *Invest Ophthalmol Vis Sci*. 2014; 55: 1867. 24576872 10.1167/iovs.13-13303

[23] Cimalla P, Walther J, Mittasch M, Koch E. Shear flow-induced optical inhomogeneity of blood assessed in vivo and in vitro by spectral domain optical coherence tomography in the 1.3 µm wavelength range. *J Biomed Opt*. 2011; 16: 116020. 22112125 10.1117/1.3653235

[24] Muraoka Y, Tsujikawa A, Kumagai K, et al. Age- and hypertension-dependent changes in retinal vessel diameter and wall thickness: an optical coherence tomography study. *Am J Ophthalmol*. 2013; 156: 706–714. 23876868 10.1016/j.ajo.2013.05.021

[25] Rim TH, Choi YS, Kim SS, et al. Retinal vessel structure measurement using spectral-domain optical coherence tomography. *Eye (Lond)*. 2016; 30: 111–119. 26493040 10.1038/eye.2015.205 PMC4709546

[26] Zhu TP, Tong YH, Zhan HJ, Ma J. Update on retinal vessel structure measurement with spectral-domain optical coherence tomography. *Microvasc Res*. 2014; 95: 7–14. 24976361 10.1016/j.mvr.2014.06.007

[27] Salvetti M, Rosei CA, Paini A, et al. Relationship of wall-to-lumen ratio of retinal arterioles with clinic and 24-hour blood pressure. *Hypertension*. 2014; 63: 1110–1115. 24516107 10.1161/HYPERTENSIONAHA.113.03004

[28] Harazny JM, Ritt M, Baleanu D, et al. Increased wall:lumen ratio of retinal arterioles in male patients with a history of a cerebrovascular event. *Hypertension*. 2007; 50: 623–629. 17698722 10.1161/HYPERTENSIONAHA.107.090779

[29] Ritt M, Harazny JM, Ott C, et al. Analysis of retinal arteriolar structure in never-treated patients with essential hypertension. *J Hypertens*. 2008; 26: 1427–1434. 18551020 10.1097/HJH.0b013e3282ffdc66

[30] Rogers S, McIntosh RL, Cheung N, et al. The prevalence of retinal vein occlusion: pooled data from population studies from the United States, Europe, Asia, and Australia. *Ophthalmology*. 2010; 117: 313–319.e311. 20022117 10.1016/j.ophtha.2009.07.017 PMC2945292

[31] Hayreh SS. Ocular vascular occlusive disorders: natural history of visual outcome. *Prog Retin Eye Res*. 2014; 41: 1–25. 24769221 10.1016/j.preteyeres.2014.04.001 PMC4073304

[32] Tomita R, Iwase T, Ueno Y, et al. Differences in blood flow between superior and inferior retinal hemispheres. *Invest Ophthalmol Vis Sci*. 2020; 61: 27. 10.1167/iovs.61.5.27 PMC7405729 32421146

[33] Yu PK, Tan PE, Morgan WH, Cringle SJ, McAllister IL, Yu DY. Age-related changes in venous endothelial phenotype at human retinal artery-vein crossing points. *Invest Ophthalmol Vis Sci*. 2012; 53: 1108–1116. 22273724 10.1167/iovs.11-8865

[34] Jefferies P, Clemett R, Day T. An anatomical study of retinal arteriovenous crossings and their role in the pathogenesis of retinal branch vein occlusions. *Aust N Z J Ophthalmol*. 1993; 21: 213–217. 8148137 10.1111/j.1442-9071.1993.tb00959.x

[35] Gouliopoulos N, Siasos G, Moschos MM, et al. Endothelial dysfunction and impaired arterial wall properties in patients with retinal vein occlusion. *Vasc Med*. 2020; 25: 302–308. 32308146 10.1177/1358863X20913609

[36] Wu CY, Riangwiwat T, Limpruttidham N, Rattanawong P, Rosen RB, Deobhakta A. Association of retinal vein occlusion with cardiovascular events and mortality: a systematic review and meta-analysis. *Retina*. 2019; 39: 1635–1645. 30829987 10.1097/IAE.0000000000002472

[37] Li M, Hu X, Huang J, Tan Y, Yang B, Tang Z. Impact of retinal vein occlusion on stroke incidence: a meta-analysis. *J Am Heart Assoc*. 2016; 5(12): e004703. 28007745 10.1161/JAHA.116.004703 PMC5210429

[38] Szigeti A, Schneider M, Ecsedy M, Nagy ZZ, Récsán Z. Association between retinal vein occlusion, axial length and vitreous chamber depth measured by optical low coherence reflectometry. *BMC Ophthalmol*. 2015; 15: 45. 25925557 10.1186/s12886-015-0031-1 PMC4423091

[39] Aritürk N, Oge Y, Erkan D, Süllü Y, Mohajerý F. Relation between retinal vein occlusions and axial length. *Br J Ophthalmol*. 1996; 80: 633–636. 8795376 10.1136/bjo.80.7.633 PMC505558

[40] Goldstein M, Leibovitch I, Varssano D, Rothkoff L, Feitt N, Loewenstein A. Axial length, refractive error, and keratometry in patients with branch retinal vein occlusion. *Eur J Ophthalmol*. 2004; 14: 37–39. 15005583 10.1177/112067210401400106

[41] Salmon AE, Sajdak BS, Atry F, Carroll J. Axial scaling is independent of ocular magnification in OCT images. *Invest Ophthalmol Vis Sci*. 2018; 59: 3037–3040. 30025118 10.1167/iovs.17-23549 PMC6005622

