# Supplementary material for: Narrowing Ratio of Retinal Veins at Arteriovenous Crossing in Patients With Branch Retinal Vein Occlusion Versus That in Healthy Individuals
Source: Invest Ophthalmol Vis Sci. 2023 Nov 16;64(14):22. doi: 10.1167/iovs.64.14.22 (PMC10664725; doi:10.1167/iovs.64.14.22)
Supplement: Supplement 1 [file iovs-64-14-22_s001.pdf]

SUPPLEMENTARY MATERIALS

Table S1. Clinical characteristics of BRVO eyes.

BRVO eyes (n = 48)	
Duration from onset (month)	27.1 ± 23.1
Presence of an ischemia area (presence/absence, eyes)	22 / 26
Type of BRVO (major BRVO/macular BRVO, eyes)	29 / 19
Past treatment	
Retinal laser photocoagulation (eyes)	20
Intravitreal injection of anti-VEGF agent (eyes)	36
Sub-tenon injection of triamcinolone acetonide (eyes)	5

BRVO, branch retinal vein occlusion; VEGF, vascular endothelial growth factor.

Table S2. Parameters of the vessels measured by optical coherence tomography.

	All groups	Young group	Control group	Fellow eye group	BRVO eye group	*P* value
Calculated internal diameter of the artery (μm)	79.0 ± 17.5	80.8 ± 14.9	77.4 ± 17.2	77.6 ± 18.0	81.2 ± 18.6	0.522
Arterial wall–lumen ratio	0.355 ± 0.078	0.321 ± 0.068	0.356 ± 0.071	0.368 ± 0.083	0.360 ± 0.081	0.010
Calculated internal diameter of the vein (μm)	107 ± 23.1	106 ± 19.0	103 ± 26.3	108 ± 19.9	110 ± 24.7	0.100
Actual internal diameter of the vein (μm)	81.7 ± 20.6	89.6 ± 16.7	80.5 ± 21.8	79.6 ± 20.4	80.4 ± 20.7	0.020

BRVO, branch retinal vein occlusion.
